# Randomised controlled trial of HOYA one-day multifocal contact lenses: The HOMCL trial

**DOI:** 10.1016/j.heliyon.2024.e40137

**Published:** 2024-11-08

**Authors:** Bruce JW. Evans, Robert Yammouni, Fabrizio Zeri, Silvia Tavazzi, Giulia Carlotta Rizzo, Bo Lauenborg, Rasmus Hagenau, Amanda Wittendorf, Manuela Eckert Andersen, Dimple Shah, Natalia Vlasak

**Affiliations:** a Institute of Optometry, 56-62 Newington Causeway, London, SE1 6DS, UK; b Department of Optometry and Visual Sciences, School of Health and Psychological Sciences, City St George's, University of London, London, EC1V 0HB, UK; c Department of Materials Science, University of Milano-Bicocca, Via Roberto Cozzi, 55, 20125, Milan, Italy; d COMiB Research Centre in Optics and Optometry, University of Milano-Bicocca, Via Roberto Cozzi, 55, 20125, Milan, Italy; e College of Health and Life Sciences, Aston University, Birmingham, B4 7ET, UK; f Kontaktlinse Instituttet, Ferdinand Sallings Stræde 6-18, 8000, Århus C, Denmark; g HOYA Vision Care, Radarweg 29, 1043 NX, Amsterdam, the Netherlands

## Abstract

**Trial design:**

Double-masked crossover RCT (Research Registry: #8136) comparison of a new HOYA one-day disposable multifocal contact lens (HOMCL) with Alcon DAILIES TOTAL1® Multifocal (ADT1).

**Methods:**

Sixty presbyopic participants from three countries attended for baseline measurements and fitting of both lens types and then for a fortnight completed daily diaries of symptoms with habitual optical correction, and VF-14 questionnaire. Participants collected either HOMCL or ADT1, when the vision was measured again, and they wore this product for a fortnight, completing daily diaries and VF-14. Participants then collected the other type and had vision and symptoms recorded in the same way.

**Results:**

There were no serious adverse events. Primary outcomes were no significant differences between the lens types in willingness to purchase nor stated preference; no significant differences in the daily symptom ratings of comfort; statistically significant findings with the daily symptom ratings and VF-14 of better near vision with HOMCL and better distance vision with ADT1. Secondary outcomes were better high contrast distance visual acuity with ADT1 (<1 line), better low contrast distance visual acuity with ADT1; faster Wilkins rate of reading test with HOMCL; no significant differences in near visual acuity, wearing time, or number of lenses required during fitting process. Exploratory analyses were better handling scores with ADT1; and some, but not all of the dry eye data indicating better acceptance of HOMCL by patients with relatively dry eyes. For all measures, there were some participants who preferred/performed better with each lens type.

**Conclusions:**

The differences between the performance of the two products were small. There was a trend in some clinical measurements and the daily diary data and VF-14 questionnaire, for HOMCL to outperform ADT1 for near vision and vice versa for distance vision.

## 1 Introduction

Over the last 45 years, various brands of soft multifocal contact lenses (MFCL) have been available. Typically, they have a progressive addition lens design, most commonly with the central part of the lens focussed for near vision, the lens periphery focussed for distance vision, and the power gradually changing between the two zones [1].

With progressive addition lens spectacles, the eye moves so its visual axis intersects the portion of the lens with the required power for a given viewing distance. In contrast, with MFCL the contact lenses (CL) move with the eye and therefore there will always be an out-of-focus image in addition to an in-focus image. This particular way of functioning, called simultaneous vision [2], represents a fundamental limitation of MFCL [3,4]. Different optical designs of MFCLs have evolved to minimise this limitation and maximise performance [4]. However, it is still difficult to predict the success of MFCL fitting [5] and although the success rate at first prescribing ranges from 67 to 83 %, there is a drop to 30–40 % for long-term wearers [6].

There are several factors that make it difficult to predict the success of MFCL and to undertake research in this topic. The first challenge is discrepancies between psychophysical responses measured by practitioners and patient satisfaction. For example, distance and near visual acuities can be a poor indicator of whether a patient is content with CL [5]. The term ‘20/happy’ has been used with MFCL because the corrected vision with MFCL may be reduced compared with spectacle acuity but the patient is satisfied. Conversely, some patients exhibit reasonable visual acuities in the clinic but are dissatisfied in everyday life. Recently, it has been shown that the subjective overall vision satisfaction at the time of dispensing is strongly linked to the intention of purchasing after 1 week of wear [7].

A second point is that when determining the optimal prescription of MFCL for a patient, the prescription for spectacles is only a starting point and a customised approach is required [6]. For some patients, the first lens power choice indicated by the manufacturer's fitting guide will be optimal, but often this is not the case. The fitting guide must be followed to select a second choice of power to determine if this better meets the wearer's expectations. In some cases, a third parameter change is required. It is unusual to require more than three options.

A third point concerns the time to wait after the fitting to assess the result. The usual procedure [6] is to allow a minimum of 15–20 min for the lenses to settle prior to the evaluation of lens performance but this varies in different studies. Finally, it should be considered that with MFCL, visual performance at lens dispensing is not representative of performance after 4 days [8,9] or up to 15 days of wear [10,11]. Changes in brain response to MFCL has been demonstrated since the first stages of wear [12], although a review concluded that adaptation to MFCL may require two weeks of wear [6].

The purpose of the present study is to evaluate the performance of a new product, the HOYA one-day MFCL (HOMCL). This is a centre-near simultaneous vision MFCL made of the same silicone hydrogel material as the HOYA single vision one-day disposable CL (sorafilcon A), which attained good acceptance in an evaluation with experienced CL wearers [13]. HOMCL has been designed to optimise performance and in the present research is compared with a leading brand of one-day centre-near MFCL, Alcon Dailies Total 1 (ADT1).

## 2 Methods

### 2.1 Study design

**Fig. 1:**
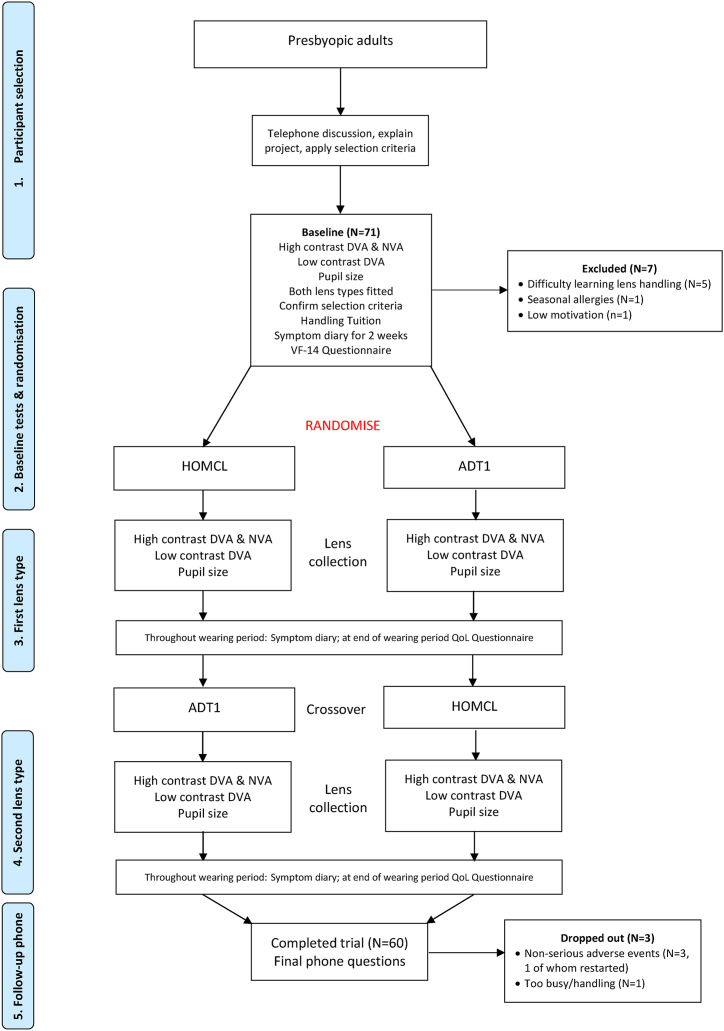
Prisma flow chart of study design and participant numbers. DVA, distance visual acuity; NVA, near visual acuity; CS, contrast sensitivity; WRRT, Wilkins Rate of Reading Test.

The study is a prospective double-masked randomised crossover trial following the principles outlined in the CONSORT statement relating to crossover RCTs [14]. The protocol is summarised in the Prisma flow chart in Fig. 1. Each type of lens was worn for a fortnight and the main outcome measures were obtained from participant ratings using daily diaries and a questionnaire completed at the end of the two week wearing periods.

The initial plan was to adopt a non-inferiority design, but the authors became aware of reports in the literature of significant methodological issues with non-inferiority designs [[15], [16], [17]], and with another approach that was considered, equivalence designs [[18], [19], [20]]. For example, non-inferiority trials require researchers to determine a non-inferiority margin [15,17,20], which involves assumptions [[19], [20], [21]] which may not be correct [17,22]. Other methodological issues may inflate the risk of establishing non-inferiority [[23], [24], [25], [26], [27], [28]]. A 2024 systematic review of methodological quality in non-inferiority trials concluded that “the non-inferiority trial design is inherently susceptible to bias despite adherence to CONSORT recommendations” [15]. Another review questions the clinical utility of the dichotomy between non-inferiority and superiority designs, arguing for patient-centric results [17]. Crucially, Ranganathan et al. (2022) highlighted that non-inferiority and equivalence designs are only appropriate when the control has already been proven to be superior to a placebo under similar conditions [18]. This criterion does not appear to have been met for the control intervention (ADT1) in the present work (a PubMed search reveals no trials comparing ADT1 with an inert placebo).

Whilst considering this emerging literature on the limitations of non-inferiority designs, the authors were increasingly impressed with a feature of the move to transparent data reporting, whereby full data are presented graphically rather than summary statistics [[29], [30], [31]]. Therefore, the final decision on study design and approach to analysis was to adopt a conventional superiority design but wherever practical to present data with full transparency, showing individual datapoints. With this approach, readers can clearly visualise the margin of overlap in performance of the two lens designs, and make their own interpretation of the clinical significance.

### 2.2 Participants

Participants were presbyopes meeting the selection criteria in Table 1. The selection criteria were deliberately broad to maximise the population to which the study findings will be relevant and to facilitate recruitment. This approach in clinical trials is described as pragmatic and improves clinical relevance and generalisability [[32], [33], [34], [35], [36], [37]]. Patients who already wore CL could participate unless they were wearing MFCL or benefitted from monovision. The reason for not including patients already wearing MFCL was to avoid the unpredictable influence of the multifocal design habitually used. This choice was reinforced by the fact that fitting MFCL for the first time is a common everyday scenario in practice. During the study, participants were asked to wear their study lenses at least 16 h a week, but this was not an inclusion criterion.

**Table 1:** Participant selection criteria.

Type of selection criterion	Criteria
Inclusion criteria	1. Aged over 40 years with symptoms of presbyopia: near vision difficulties when only the distance refractive error is corrected. 2. Wearing or considering using any form of correction of presbyopia. 3. Refractive error can be corrected by CL of both types under evaluation in the power range +2.00D to −6.00D.
Exclusion criteria	1. Currently wear or have worn within the last year MFCL of any type or are currently spectacle free from monovision. 2. Astigmatism in either or both eyes of more than 0.75DC. 3. Monocular best corrected acuity in each eye worse than 0.2 LogMAR. 4. Pseudophakia of one or both eyes. 5. Ocular pathology, systemic pathology that is likely to worsen in the timescale of the study or cause transient blur (e.g., diabetes, pregnancy) or interfere with corneal health or clarity. 6. With trial lenses of either type of CL, not possible to obtain a fit and/or vision that the patient finds acceptable and/or meets legal requirements for driving if the patient is a driver. 7. Anticipate being unable to attend aftercare in the next three months.

The use of a crossover design means each participant acts as their own control. This avoids inter-subject variation and increases statistical power. Therefore, compared with a parallel group trial fewer participants are required for the study to be powered to detect a given magnitude of difference in performance between the two products [38].

To estimate the sample size, a priori power analysis was performed using the program G∗Power (version 3.1.9.7) [39]. The key primary endpoint was participants’ choice, when they finish the trial, as to which brand they prefer. For this, a sign test is appropriate and if the study is to be powered to detect a medium effect size (g = 0.30) with 95 % power and for a p-value of 0.05 [39] 49 participants were required. This figure is in good agreement with the sample size of recent research on MFCL which studied populations of 55∗ [11], 49∗ [40], 16 [41], 25∗ [42], 38∗ [43], and 32∗ [44]. Although none of these publications included a sample size calculation, all five studies marked with an asterisk found statistically significant differences in the comparisons of lens performance, suggesting that sample sizes of 25 or more are likely to be adequate to detect statistically significant differences. The study recruited 60 participants to safely exceed this requirement in case of missing data.

### 2.3 Materials

The novel MFCL in this study is a silicone hydrogel daily disposable made by HOYA in sorafilcon A material (HOMCL). This lens has a back optic zone radius of 8.6 mm, a total diameter of 14.2 mm, equilibrium water content of 48 % and Dk/t of 124 Fatt units (at −3.00 DS). Its optical design is centre-near. The comparison lens is a leading brand of MFCL, Alcon DAILIES TOTAL1® multifocal (Alcon Laboratories, Fort Worth, TX, USA) (ADT1), a daily disposable centre-near CL. It is made in delefilcon A material, with a back optic zone radius of 8.5 mm, total diameter of 14.1 mm, core equilibrium water content of 33 % and Dk/t of 156 Fatt units (at −3.00 DS). Both lens types are available in three addition powers, +1.00, +1.50, and +2.00 for HOMCL and low, medium, and high for ADT1.

### 2.4 Procedure

The research followed the tenets of the Declaration of Helsinki [45], and underwent favourable ethical review from Ethics Committees at all three sites (reference numbers & dates: London, 22/BE/0101, Jul 9, 2022; Milan, 0110484, Sep 26, 2022; Aarhus, 2301179, Apr 14, 2023). Written informed consent was obtained from all participants. An adverse event reporting system was adopted which used open questions to facilitate the detection of non-prespecified harms. Participants were given a gift voucher (£25 or equivalent) as a token of appreciation and contribution towards travel costs.

Potential participants were sought using publicity and advertisements. Investigators contacted potential participants for a preliminary discussion about the project and to screen for suitability. Suitable potential participants were sent a Participant Information Sheet (PIS) and consent form and arranged a baseline assessment appointment. The PIS required an eye examination within the last two years and stressed that the research does not replace the need for routine eyecare.

#### 2.4.1 Preliminary phase: baseline assessment

The clinical procedures carried out in this phase are summarised in Table 2. For the tests numbered 4–9, tests at baseline were undertaken whilst participants wore the refractive correction found in subjective refraction at the baseline appointment in an optometric trial frame. At follow-up, these tests were carried out with the MFCL.

**Table 2:** Clinical procedures at baseline and follow-up assessments. D, distance; N, near; VA, visual acuity.

Order	Test/procedure	Details
1	Symptoms & history	Including note of prescription & type of refractive correction used. OSDI questionnaire for dry eye.
2	Biomicroscopy	Full anterior segment biomicroscope examination [46] using Efron grading scales [[47], [48], [49]]. Non-invasive tear break-up time, fluorescein tear break-up time, tear meniscus height, and Meibomian gland assessment (biomicroscopy with transillumination, using Pult's Meiboscale) [50].
3	Refraction	Subjective refraction (non-cycloplegic)
4	High contrast D VA	Best corrected monocular and binocular high contrast visual acuities (letter-by-letter scoring) at distance (computerised chart, Sloan ETDRS letters).
5	High contrast N VA	Best corrected monocular and binocular high contrast visual acuities (letter-by-letter scoring) [25] at near (40 cm, LogMAR chart).
6	Low contrast D VA	Best corrected monocular and binocular low (12.5 %) contrast visual acuities (letter-by-letter scoring) [25] at distance (computerised chart, Sloan ETDRS letters).
7	Near contrast sensitivity	Hamilton Veale contrast sensitivity test binocularly.
8	Stereopsis	Randot Mk 2 random dot shapes and contoured circles. Tested & scored according to the manufacturer instructions: “Record the level of stereopsis at the last one chosen correctly”.
9	Wilkins Rate of Reading Test (WRRT) [51]	Tested according to the designer's instruction [52], with one exception. At each visit, this test was carried out twice and the mean performance calculated from these two to avoid a possible learning effect [53]. At baseline, this was undertaken with participants wearing their subjective near refractive findings. At the two CL collect visits, the WRRT were undertaken (again, twice each time) with the participant wearing MFCL of the type they were collecting. The test was used with versions in the local language of the sites [53].
10	Pupil size	Pupil size was measured under photopic and scotopic conditions with topographer pupillography function. In the UK, the Keratron Scout (Optikon 2000 SpA, Rome, Italy) was used. In Italy and Denmark, the Sirius+ (CSO, Florence, Italy) was used, and measurements under mesopic conditions were obtained in addition to photopic & scotopic.

At the baseline appointment, after the tests in Table 2, both types of CL were fitted using each manufacturer's fitting guide to: choose the first pair of trial lenses; use the recommended settling time; follow manufacturer's advice on checking the fit and over-refraction; select and check, if necessary, a second, third, etc pair of lenses until the optimal lens pair was determined for each lens type. The fitting guidelines that were used for HOMCL are available as Supplementary Material File 1. The fitting guidelines that were used for ADT1 were those in use in the relevant country at the time of the trial.

The number of lens changes during the fitting process was recorded as an outcome variable. If, for either type of lens, it was not possible to obtain satisfactory vision or fit with any trial lenses, the participant was excluded from the research. By “satisfactory”, it is meant that the lenses fitted in a clinically acceptable way [54], and gave vision that the participant found acceptable and which met the legal requirements for driving in that jurisdiction, if the participant drives. New participants were started in place of any excluded for this reason.

New CL wearers were taught lens application and removal using single vision one-day disposable soft CL of another brand. At the collection appointments, participants applied lenses out of sight of the researcher, to maintain the mask. All participants were give verbal and written information on handling, safe CL wear and emergency procedures in case of adverse events.

Participants were shown how to complete the daily symptom diary (see Supplementary Material), and VF-14 questionnaire [[55], [56], [57], [58]] (see Supplementary Material) and the importance of completing this information was stressed at all appointments. Participants were instructed to start completing the (baseline) daily diary on the next day and to complete the baseline VF-14 in a fortnight, after they have finished the diary. It was explained that completion of both these instruments must refer to the situation whilst wearing their habitual refractive correction.

Further appointments were booked, avoiding holidays so that as far as possible participants carried out similar activities during each wearing period.

#### 2.4.2 “CL study phase”: procedure after baseline assessment

A researcher sent each participant reminders by email or text to complete the daily symptom diary, with reminders on 1st, 3rd, 6th, 10th, 14th day of each period. On the 14th day of each period, the reminder included to complete the VF-14 questionnaire. Random allocation codes were created for each centre using www.sealedenvelope.com using blocks of four, so that half of the 20 participants in each centre would start with each type of CL. Labels were placed over blister packs to disguise the lens identity.

At the first collection appointment, the participant inserted the first pair of the first type of lenses. After the lenses had settled for 20 min, the investigator repeated tests 4–9 in Table 2 with the participant wearing the first type of lenses. The researcher instructed participants that they should wear these lenses every day, unless there is a problem in which case contact the researcher. Also, that they should complete the diaries and questionnaires, referring to the situation with the MFCL that they were wearing at this time. The participant was reminded to restart the diaries on the first day of wear, and was reminded regularly, as detailed above. On the 14th day with the first lens type, the participant received the final reminder about the diary and was reminded about the VF-14 questionnaire and about the forthcoming appointment.

In view of the minimum physiological changes from short-term soft CL wear, there was no requirement for a prolonged washout period between wearing the two lens types. The minimum required washout period was overnight.

At the second collection appointment, the investigator started by asking the following questions.

a. “Please think about the lenses you have worn over the last fortnight. If they were available at a price you found affordable, would you wish to continue wearing them?
b. Please think about how easy the lenses were to handle. On a scale of 1–10, when 1 is impossible to handle and 10 is incredibly easy to handle, what score would you give them?
c. Do you have any other comments about the lenses you wish to make?”

The participant inserted a pair of the second type of lenses. After the lenses had settled for 20 min, the investigator repeated tests 4–9 in Table 2 with the participant wearing these lenses. The participant was reminded to restart the diaries, and was reminded regularly, as detailed above. On the 14th day with the second lens type, the participant received the final reminder about the diary and was reminded about the VF-14 questionnaire and a time was agreed for the telephone follow-up.

As soon as possible after the participant was due to have finished the trial, the investigator telephoned the participant, checked completion of the trial and the final VF-14, and asked the following questions, noting the answers.

a. Please think just about the lenses you have worn over the last fortnight. If they were available at a price, you found affordable, would you wish to continue wearing them?
b. Please think about how easy the lenses were to handle. On a scale of 1–10, when 1 is impossible to handle and 10 is incredibly easy to handle, what score would you give them?
c. Do you have any other comments about the lenses you wish to make?
d. Finally, please recall, as best you can, the previous type of lenses you wore for the first fortnight of the trial. Think about the performance of the first type and the performance of the second type. If they were the same price, which pair would you prefer, the first or the second, or are you unable to choose?”

Investigators were not masked to the identity of the lenses at the fitting stage, so that they could follow the manufacturers’ fitting guidelines. After the fitting, the investigator sent to HOYA, the Sponsor, the specification of the optimally fitting lens for each type and from then onwards the investigator was masked as to the identity of the lenses. Like the participant, the investigator did not know which type of lens was worn first. This was achieved by a researcher who did not see participants covering the blister packs (after checking that HOYA had provided the correct specification) with labels that only identified right/left lens and participant number and following the randomisation to control which brand was worn first. The study was therefore double-masked.

### 2.5 Outcomes and statistical analysis

The researcher (BE) who broke the code for which lens type each participant had worn first was different to the researcher undertaking the statistical analysis (RY). BE used pseudonyms to replace brand names, so that RY analysed the data without knowing the identity of the brands. The code was only broken after the data analysis was complete.

The primary outcomes were established before data collection was started and pre-registered at the Research Registry (#8136; registration date July 27, 2022). These are summarised in Table 3. For each outcome, the null hypothesis is that there is no significant difference between the two brands. All data were tested for normality and parametric or non-parametric statistical tests were used as appropriate. In addition to the above primary outcomes, the secondary outcomes in Table 3 were investigated.

**Table 3:** Primary and secondary outcomes.

Type of outcome	Outcome
**Primary outcomes**	Participant ratings, based on daily diaries of symptoms and visual performance. Participant ratings, based on VF-14 diaries of symptoms and visual performance. Proportion of participants who say, after each fortnight, that they would purchase that lens type. Participant preference in final question at end of trial.
**Secondary outcomes**	Proportion of people attending baseline fitting who could be successfully fitted with each lens type High contrast binocular distance visual acuity Low contrast binocular distance visual acuity High contrast binocular near visual acuity Near vision contrast sensitivity Stereopsis (Randot shapes and Randot circles) Rate of reading (WRRT) Is there any statistically significant difference in the wearing time with each type of lens? If there are any adverse events, which lens type did they occur with and can any conclusions about lens performance be made? Does participant pupil size impact on performance and, if so, does this differ for the two brands? Is there a difference between the two lens types in the mean number of fitting lenses required?

## 3 Results

### 3.1 Attrition, adverse events, washout period

Recruitment ran from September 2022 until August 2023 and follow-up was completed in October 2023 when the required number of participants had been reached. Seven participants attended the baseline and fitting appointment and did not continue in the trial. The most common reason (5 participants) for not continuing past the baseline appointment was difficulty learning how to handle CLs. One participant was not able to wear owing to seasonal allergies. One had low motivation.

In patients who started the trial, there were three adverse events, all non-serious. One patient (when wearing HOMCL) had a foreign body trapped under a CL causing discomfort, another had an episode of uveitis unrelated to CL wear (wearing HOMCL) and the third reported discomfort for no apparent reason (wearing ADT1). The first two participants abandoned the trial and the third, after discontinuing CL wear for a few days, restarted the trial. A fourth participant discontinued the trial because they were too busy/considered it took too long to apply the lenses in the morning (they were offered further tuition in lens handling but declined). As per the protocol, for all participants who did not proceed beyond the fitting appointment and for the two patients who discontinued after the fitting appointment, other participants were started in their place, so that 60 participants completed the trial.

The minimum washout period was overnight and the median was 6 days. All participants wore the lenses they were instructed to wear in the relevant period and therefore actual groups did not differ from original assigned groups.

### 3.2 Study population

The study population is summarised in Table 4. Thirty-nine (65 %) of participants were females. For all these descriptive data, there were no significant differences between the participants who started with HOMCL and those who started with ADT1 (p > 0.15). For ADT1, 18.3 % of add powers were low, 41.7 % medium, and 40.0 % high. For HOMCL, 43.3 % were +1.00, 31.7 % were +1.50, and 25.0 % were +2.00.

**Table 4:** Summary of study population. The second column is median unless otherwise stated.

**Variable**		**Median/(mean)**	**SD**	**Minimum**	**Maximum**
Age (years)		55.9 (mean)	7.8	41.9	74.8
Spherical equivalent refraction (Dioptres)	Right eye	−1.25		−5.50	+2.38
	Left eye	−1.44		−5.75	+2.25
Astigmatism (Dioptres)	Right eye	0.50		0.00	0.75
	Left eye	0.50		0.00	0.75
Near addition (Dioptres)	Right eye	2.00		0.75	2.50
	Left eye	2.00		0.75	2.50

### 3.3 Primary outcomes

The first primary outcome is participants' ratings from the daily diaries. Daily diaries (Supplementary Material) used visual analogue scales, with results scored from 0 to 100, with higher scores signifying better performance. For each individual question in the diary, the participant's mean score was calculated from all the diaries completed for a given intervention. As noted in the methods, frequent reminders were used to encourage compliance and there were very few (<2 %) missing diary entries. This is considered further in the discussion. The diary data are summarised in Table 5 and are described in more detail, including violin plots, later.

**Table 5:** Summary of results (median, inter-quartile range, and Wilcoxon signed ranks test; N = 60) on symptoms of visual performance and comfort from the daily diaries. Higher ratings indicate better performance.

Rating	ADT1	HOMCL	p-value
Distance vision	82.0 (67.6–89.9)	72.8 (61.3–83.2)	0.002
Intermediate vision	73.1 (49.1–85.2)	77.3 (62.3–83.8)	0.073
Near vision	65.3 (42.1–82.6)	71.5 (59.5–82.6)	0.001
Comfort	83.6 (69.4–90.5)	82.7 (71.2–88.6)	0.74

The second primary outcome relates to the VF-14 questionnaire (Supplementary Material). The mean response to all 14 questions was calculated and the results compared for the two lens types. The response was not significantly different (paired *t*-test, p = 0.42) for ADT1 (mean 0.97, SD 0.63) and HOMCL (mean 0.91, SD 0.61). Results of each item in the VF-14 are summarised in Table 6.

**Table 6:** Results for single items of VF-14 questionnaire (N = 60). The results are median and range, and Wilcoxon signed ranks test. Lower ratings indicate better performance.

Question	HOMCL	ADT1	p-value
Small print	1 (0–4)	2 (0–4)	<0.001 HOMCL better
Reading books	1 (0–3)	2 (0–4)	0.002 HOMCL better
Large print books	0 (0–3)	1 (0–3)	0.019 HOMCL better
Faces that are close	0 (0–3)	0 (0–2)	1.00
Stairs, curbs, etc	0 (0–3)	0 (0–3)	1.00
Traffic signs, street signs	1 (0–4)	1 (0–3)	<0.001 ADT1 better
Fine handcrafts, sewing, etc	1 (0–4)	2 (0–4)	0.28
Writing cheques, filling forms	1 (0–4)	1 (0–4)	0.008 HOMCL better
Board games	0 (0–3)	0 (0–2)	0.71
Sports	1 (0–3)	0 (0–2)	0.053 ADT1 better
Cooking	0 (0–3)	0 (0–2)	0.20
Watching TV	1 (0–3)	1 (0–3)	0.15
Day driving	1 (0–4)	0 (0–4)	0.048 ADT1 better
Night driving	2 (0–4)	1 (0–4)	0.010 ADT1 better

The third primary outcome was the proportion who at the end of a fortnight's wear said that if the lenses were available at a price they found affordable they would continue to use them. The proportion who would buy ADT1 after wearing for a fortnight (30/60) was not significantly different (chi-square, p = 0.58) from the proportion who would buy HOMCL after wearing for a fortnight (27/60).

For the fourth primary outcome, participant preference for ADT1 versus HOMCL in the final question at the end of the trial, six participants (10 %) could not choose. Of the 54 participants who expressed a preference, 31 (57 %) preferred ADT1 and 23 (43 %) preferred HOMCL. For those that could choose, the difference in preference was not significantly different to chance (sign test, p = 0.34).

### 3.4 Secondary outcomes

The secondary outcomes are summarised in Table 7. The stereopsis test (Randot 2) includes two subtests, the main circles test, which is contoured and has 10 levels, and the shapes test, which employs a random dot design and has three levels [59]. All three centres (N = 60) used the circles test and these results are reported in Table 7. Additionally, two of the three centres (London and Milan) also used the shapes test and for these data (N = 40) there was significantly better performance (Wilcoxon signed ranks test, p = 0.014) with HOMCL (log arc sec; median 2.00, range 2.00–2.60) than with ADT1 (median 2.30, range 2.00–2.70).

**Table 7:** Results for secondary outcomes. For all tests, N = 60 unless otherwise stated. ISTT, independent samples t-test; LogMAR, log of minimum angle of resolution; med., median; PSTT, paired samples *t*-test; SD, standard deviation; WRRT, Wilkins rate of reading test; WSRT, Wilcoxon signed ranks test.

Outcome measure	Statistics
Proportion of people attending baseline fitting who could be successfully fitted with each lens type	There were no participants whose fitting had to be abandoned for reasons of fit or vision.
High contrast binocular distance visual acuity (LogMAR) (see Fig. 2)	ADT1 (med. −0.08, range −0.20 to 0.16, IQR -0.14 to 0.00) better (WSRT p=<0.001) than HOMCL (med. −0.005, range −0.28 to 0.16, IQR -0.08 to 0.03)
Low contrast binocular distance visual acuity (to control for possible calibration differences, scores in each centre were converted to z-scores and these pooled and analysed by ranks)	ADT1 (med. 10.5, range 1–40, IQR 1 to 16) better (WSRT p < 0.001) than HOMCL (med. 16.0, range 1–40, IQR 4 to 25)
High contrast binocular near visual acuity (LogMAR) (see Fig. 2)	ADT1 (mean 0.054, SD 0.13) not significantly different (PSTT p = 0.31) to HOMCL (mean 0.033, SD 0.10)
Near vision contrast sensitivity (logCS)	ADT1 (med. 1.80, range 1.50–1.95, IQR 1.80 to 1.95) not significantly different (WSRT p = 0.22) to HOMCL (med. 1.84, range 1.65–1.95, IQR 1.80 to 1.95)
Stereopsis (Randot circles; log arcSec))	ADT1 (med. 2.00, range 1.30–2.60, IQR 1.70 to 2.11) not significantly different (WSRT p = 0.17) to HOMCL (med. 1.80, range 1.20–2.30, IQR 1.60 to 2.00)
Rate of reading (WRRT; words per minute)	ADT1 (mean 154.0, SD 29.7) worse (PTT p = 0.023) than HOMCL (mean 160.0, SD 27.3)
Is there any statistically significant difference in the wearing time (hours) with each type of lens?	ADT1 (med. 7.3, range 3–16, IQR 4.4 to 10.4) not significantly different (WSRT p = 0.075) to HOMCL (med. 8.45 range 2.5–16, IQR 4.50 to 10.7)
If there are any adverse events, which lens type did they occur with and can any conclusions about lens performance be made?	Only 3 adverse events, one with ADT1 and two with HOMCL. All were non-serious.
Does participant pupil size impact on performance and, if so, does this differ for the two brands?	When participants preferring HOMCL were compared to those preferring ADT1, no significant difference in photopic or scotopic pupil size, but those preferring HOMCL had slightly smaller (ISTT p = 0.041, N = 34) mesopic pupil size (mean 4.55 mm, SD 0.63) than those preferring ADT1 (mean 5.03 mm, SD 0.70).
Is there a difference between the two lens types in the number of fitting lenses required? (researchers were not masked for this outcome)	ADT1 (med. 2, range 1–5, IQR 1 to 2) not significantly different (WSRT p = 0.95) to HOMCL (med. 2, range 1–4, IQR 1 to 2)

**Fig. 2:**
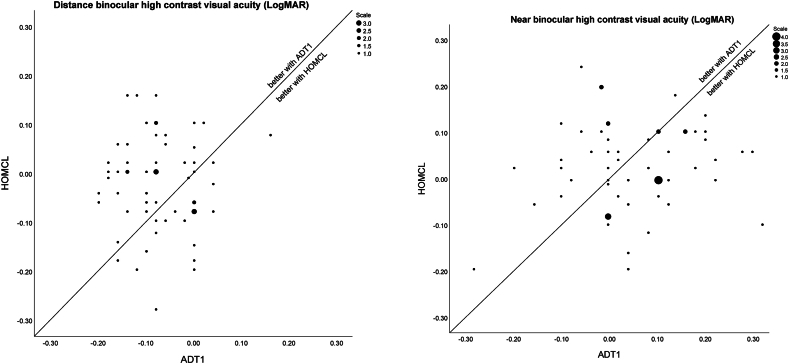
High contrast visual acuity (LogMAR) results for distance (left) and near (right). Each point is a participant, except where there are coincident points which are indicated by larger dots, as indicated in the scale. With the LogMAR units, lower values indicate better performance. Therefore, points lying above the diagonals indicate better performance with ADT1 and points below the diagonals indicate better performance with HOMCL.

High contrast visual acuity results with the two lens types are illustrated with scatterplots in Fig. 2, which show a trend for superior distance vision with ADT1 and better near vision with HOMCL. However, despite these trends it is notable that there are many participants who show the opposite tendency, sometimes to quite marked degrees.

### 3.5 Exploratory analyses

**Fig. 3:**
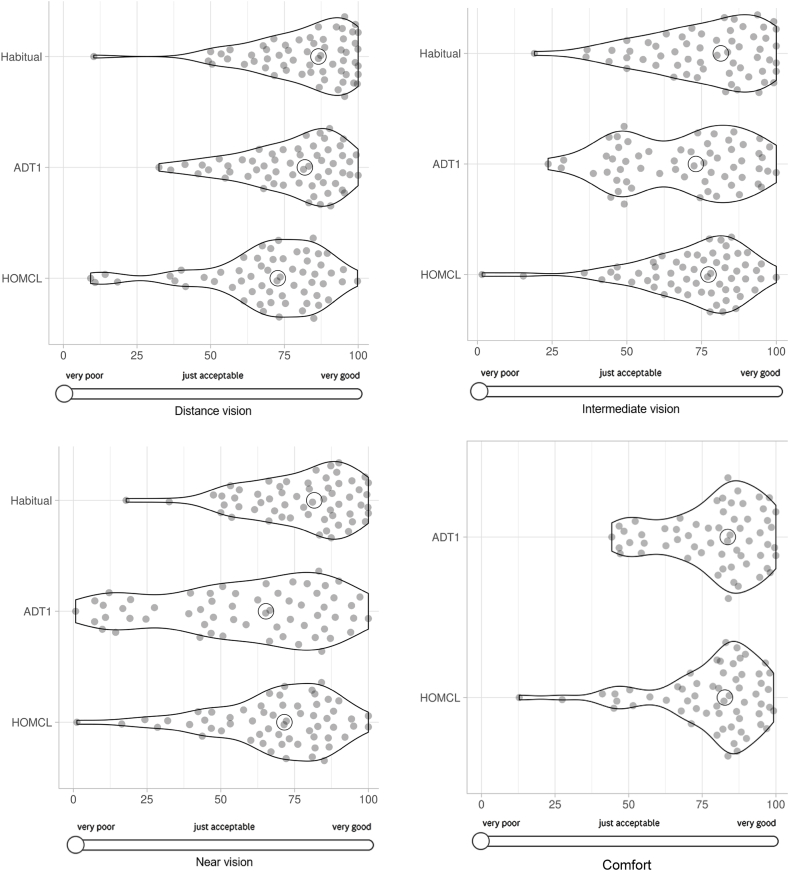
Comparison of mean daily diary ratings of distance vision, intermediate vision, and near vision (baseline v. ADT1 v. HOMCL); and comfort (ADT1 v. HOMCL). Violin plots have the advantage of showing all data, represented within each dataset by points that are pseudo-randomly spaced along the y-axis. Open circles represent the median results. The x-axis is the actual scale used in the VAS for data collection, with participants instructed to slide the circle on the left to the position on the horizontal line that reflected their symptoms each day.

During the data analysis some additional outcomes were analysed. These are unplanned and therefore best considered as exploratory. Additional outcomes relating to handling and daily diaries are reported in Table 8. The full data for the diary ratings are illustrated in Fig. 3, compared with baseline (with habitual correction) for gradings of vision.

**Table 8:** Results for additional (exploratory) outcomes relating to handling and daily diaries. Statistics are median (inter-quartile range) and p-values from Wilcoxon signed ranks test. Higher values indicate better performance.

**Outcome**	**Statistical test**	
Handling scores at end of fortnight of lens wear		ADT1: 9.0 (8–10)
		HOMCL: 8.00 (7–9)
		p=0.006
Daily diary comparison of habitual vision correction with ADT1 (full data in Fig. 3)	**Distance vision**	habitual: 86.6 (71.5–95.0)
		ADT1: 82.0 (67.6–89.9) p=0.047
	**Intermediate vision**	habitual: 81.4 (65.1–92.7)
		ADT1: 73.1 (49.1–85.2) p=0.02
	**Near vision**	habitual: 81.7 (66.3–90.0)
		ADT1: 65.3 (42.1–82.6) p<0.001
Daily diary comparison of habitual vision correction with HOMCL (full data in Fig. 3)	**Distance vision**	habitual: 86.6 (71.5–95.0)
		HOMCL: 72.8 (61.3–83.2) p<0.001
	**Intermediate vision**	habitual: 81.4 (65.1–92.7)
		HOMCL: 77.3 (62.3–83.8) p=0.092
	**Near vision**	habitual: 81.7 (66.3–90.0)
		HOMCL: 71.5 (59.5–82.6) p=0.012

Responses to the individual questions in the VF-14 questionnaire when wearing habitual optical correction compared with ADT1 and with HOMCL supported the main VF-14 head-to-head comparison in Table 6. The comparisons of ADT1 with habitual optical correction that reached statistical significance indicated more difficulty with near vision tasks with ADT1 than with habitual optical correction (p < 0.05 for small print, books, large print books, fine handcrafts and sewing, writing cheques and filling forms, playing cards). Conversely, participants had significantly more difficulty with distance vision tasks with HOMCL than with habitual correction (p < 0.005 for traffic and street signs, watching TV, daytime driving, and night-time driving). The only results that departed from this trend are that participants reported significantly (p < 0.05) more difficulty watching television and daytime driving with ADT1 than with habitual optical correction.

### 3.6 Characteristics of the participants who preferred either lens type

A chi-squared 3x2 table was constructed to investigate whether there is any relationship between lens preference (ADT1/HOMCL/could not choose) and gender. There was no significant relationship (p = 0.21).

Participants who at the end of the study expressed a preference for one lens type (N = 54) were divided into two groups: those whose final lens choice was ADT1 and those who preferred HOMCL. The mean age of participants who chose ADT1 (57.2 years, SD 8.7) was not significantly different (paired *t*-test, p = 0.31) to the mean age of participants who chose HOMCL (54.9 years, SD 7.3). Similarly, the spherical equivalent refraction and near addition did not differ significantly in the two groups (p > 0.35).

The baseline biomicroscope findings were compared in these two subgroups to determine whether there were any clinical characteristics that favoured one lens brand. The two groups did not differ significantly (p > 0.06) in Efron gradings of the following biomicroscope findings: bulbar redness, limbal redness, lid redness, lid roughness, corneal fluorescein staining, meibomian gland dysfunction, tear meniscus height, LIPCOF score. The two groups differed significantly in non-invasive tear break-up time (N = 60, p = 0.028) but not in fluorescein tear break-up time (only measured in London and Milan, N = 36, p = 0.07). Participants with a more rapid non-invasive tear break-up were more likely to prefer HOMCL than ADT1. Similarly, those with higher Meiboscale [50] scores, indicating a higher degree of Meibomian glands loss, were more likely to prefer HOMCL (p = 0.007). However, the two groups did not differ significantly in OSDI scores (p = 0.26).

### 3.7 Supplementary analyses

One of the centres was delayed in starting the study whilst awaiting ethical approval. During the data analysis it was noticed that the last 12 participants at this centre were not randomly assigned to sequence of interventions and all received HOMCL before ADT1. The double-masked design was still maintained in these cases. As a precaution, the analyses were repeated for the 48 participants who received the two lenses in random sequence (excluding the 12 who did not receive lens types randomly). This changed the findings for only one of the primary outcomes, daily diary intermediate vision for which HOMCL performed better (p = 0.025). The exploratory analyses of VF-14 data gave similar results for the first 12 questions, but for both day (p = 0.12) and night (p = 0.068) driving the two types of lenses no longer differed significantly. These supplementary analyses largely support the main findings in the full analyses.

## 4 Discussion

Adverse events were uncommon (three), mild, and occurred with both lens types. The planned analyses of the primary outcomes revealed no significant differences between the two lens types in the proportion of participants who would wish to purchase each type or in the proportion who preferred each type. There was also no significant difference between the two lens types in comfort as assessed by daily diary reports and for the overall score of the VF-14 (average of 14 questions about visual performance in a variety of tasks). However, when individual question scores were analysed for the daily diaries (Table 5) and for the VF-14 (Table 6), responses indicate significantly better performance at near vision tasks with HOMCL and for distance vision tasks with ADT1.

In the secondary outcomes, ADT1 performed significantly better than HOMCL at high and low contrast distance visual acuity. For both lens types, the median high contrast distance visual acuity was better than 6/6 (20/20), and the difference between the medians was less than one line of a standard visual acuity chart. At near, although there was a trend for HOMCL to perform better than ADT1 (supporting the daily diary and VF-14 data), the difference between the group means was equivalent to only about one letter of a typical chart and was not statistically significant. The rate of reading (WRRT) with HOMCL was statistically significantly (4 %) faster than with ADT1; although it is doubtful whether this is clinically significant. For near stereopsis, with one of the stereopsis tests the two lens types did not differ significantly, and with the other stereopsis test, HOMCL performed significantly better that ADT1.

Participants who before CL fitting had lower tear break-up time and higher Meiboscale scores (signs potentially linked to dry eye) were more likely to prefer HOMCL than ADT1. However, there was no statistically significant difference in OSDI scores between the groups preferring each lens type and no difference between the lens types in the daily diary scores for comfort.

As noted in the introduction, contemporary MFCL rely on simultaneous vision. This means that as well as in-focus rays from the object of regard that pass through the appropriate part of the lens, there must also be rays that pass through regions of the lens that are powered for other distances and therefore present out-of-focus images [4]. The different powers in the MFCLs can be provided by a progressive change in the power, radially along the surface of the optical zone of the lens. This change can be achieved with an aspheric surface either positioning the highest positive power for near vision in the centre of the lens (centre-near design) or, less commonly, with the highest positive power in the periphery of the lens (centre-distance design) [6]. In both cases, the lenses induce a certain degree of spherical aberration (pupil dependent) which can improve the depth of focus (DoF) of the system (pseudo-accommodation) but at the same time inducing a reduction of the Optical Transfer Function (OTF) [60].

**Fig. 4:**
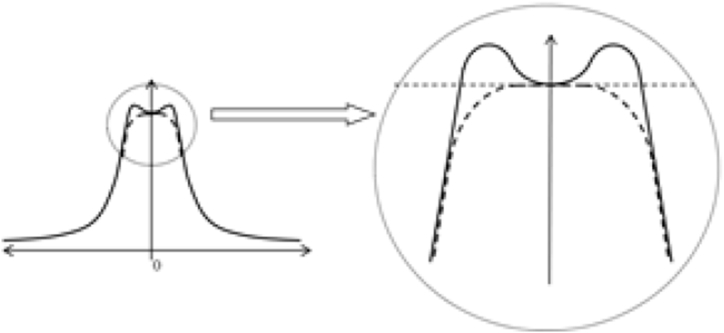
HOMCL design features to maximise performance with higher additions. Figure courtesy of HOYA.

Manufacturers strive to make improvements in new designs to improve visual performance of MFCLs. The HOMCL design uses two patented approaches to minimise two problems that are inherent in multifocal designs with higher near additions: reduced near zone size and aberrations within the near zone. The design feature illustrated in Fig. 4 aims to improve near zone stability and the lens also incorporates features to reduce aberrations.

HOMCL is made of a silicone hydrogel UV-blocking material that has been found to achieve high levels of comfort and acceptance [13]. The comparator lens, ADT1, was chosen as a market-leading well-established lens with a reputation for good visual performance and a material designed to minimise symptoms of dry eye [13,[61], [62], [63], [64]].

The maturity of the MFCL market means that, when comparing a new product with a market-leading design, it is unlikely that the new product will perform markedly better than the best available products. For any lens design, there must be a balance between the area of the lens allocated to distance vision and the zones for intermediate and near vision. The present results indicate that, although both lens types perform well for most patients at distance and near, on average HOMCL performs slightly better than ADT1 for near vision and vice versa for distance vision.

A disadvantage of descriptive statistics, and particularly measures of central tendency (mean and median) are that these can mask heterogeneity within the datasets. For example, widely different underlying datasets can produce similar bar charts [29], and therefore it is recommended that full data are presented graphically [30]. Ophthalmology and optometry researchers have been shown to underperform [31] in this movement towards transparent data reporting [29]. For this reason, the approach that has been taken for representing data graphically in this manuscript (Fig. 2, Fig. 3), illustrates individual datapoints.

This transparent data reporting reveals an important feature of MFCL data [13] that is often under emphasised. For example, considering Fig. 2 although it is clear that there is a tendency for superior performance (points above the diagonal) for distance vision with ADT1 and superior performance for near vision with HOMCL, there are many datapoints that show the opposite tendency. The same point, of many exceptions to the measure of central tendency, is apparent in the violin plots in Fig. 3. This means that it would be unsafe for a clinician to predict from the small differences in mean/median performance of the two designs that a given patient will perform better at near with HOMCL than with ADT1, or better at distance vision with ADT1 than with HOMCL. Indeed, many patients will have better distance vision with HOMCL than with ADT1 and vice versa for near vision.

Clinically, this means that the most successful clinicians will be those who are prepared to properly manage the assessment of presbyopic patients [65] by trying different brands of lenses, and who counsel their patients that they are likely to need to try more than one option to determine the brand that suits them best. A patient who is not completely happy with the performance of one design may well be satisfied on trying a different brand.

The large variation in responses between different participants illustrated in Fig. 2, Fig. 3 may be attributable, at least in part, to inter-individual variations in aberrations of the eye, especially ocular spherical aberration [4,66]. Different designs of MFCL induce varying amounts of spherical aberration [67], and ultimately optimal performance may require designs to be customised for each individual's spherical aberration [68].

### 4.1 Strengths and limitations

A strength of the study is the use of both daily diary data, providing close to real time data on symptoms, and the VF-14 standardised questionnaire, which provides data on recollected symptoms. Another strength is the use of visual analogue scales (VAS) in the daily diary. VAS are widely used in healthcare sciences [69] and one advantage, compared with Likert-type scales, is that respondents are not forced to categorize their responses, resulting in continuous variables rather than interval-level data [70]. Papas and Schultz compared VAS with numerical rating scales (NRS) and found both approaches have strengths and weaknesses [71]. A disadvantage of NRS is that participants have a tendency to round their responses [71]. VAS have been widely used in vision science [[72], [73], [74], [75]] and CL research [13,[76], [77], [78]], to grade vision [72] and ocular comfort [77,78].

It was anticipated that compliance with completing daily diaries could be a problem and this is why the importance of completing the diaries was stressed on recruitment and at all appointments and participants were sent reminders by email or text on the 1st, 3rd, 6th, 10th, and 14th day of each wearing period. No doubt these strenuous attempts to encourage compliance contributed to the very small number (<2 %) of missing diary entries. It is possible that some participants completed diaries for more than one day in batches and this is a potential limitation.

The VF-14 is a patient reported outcome questionnaire to assess vision-related quality of life, which has been used in a wide range of conditions [[79], [80], [81], [82]], including refractive error [56] and to assess the effect of CL [58]. Recently (since our study was completed) Albero-Ros and colleagues described an extensive item bank of 575 questions for evaluating MFCL [83]. This bank has yet to be evaluated on a substantial group of MFCL wearers and refined into a useable scale.

The adoption of a crossover trial design represents both a strength (the effect of noisier between-subject variance is minimised to concentrate on within-subject variance) and a possible limitation if there are order effects or an insufficient washout period. The departure from protocol in not following the randomisation sequence for 12 participants in one centre is a limitation, although masking was still maintained. Reassuringly, when the data analyses were repeated just for the 48 participants who were randomised, the main findings were supported.

The decision to concentrate on participants who are new to MFCL is both a strength and a limitation. An advantage of this approach is that it means the results are directly applicable to patients who are considering wearing MFCL for the first time, supporting generalisability to this population. A disadvantage is that adaptation to MFCLs can improve the performance of these lenses and it is possible that some participants had not reached complete perceptual adaptation to MFCLs with the first pair of lenses. However, the adoption of a crossover design would limit the impact of any such effect.

The ADT1 fitting guide was followed to the point of dispensing trial lenses, but the guide recommends after dispensing trial lenses that the “wearer now needs to experience ‘real-world’ vision for approximately one to two weeks”. Participants were not re-evaluated after one to two weeks and it is possible that for some wearers a further change to the prescription at this time may have resulted in increased performance. This also may be the case for HOMCL and therefore it is possible that the trial has underestimated real-world performance.

## 5 Conclusions

Both products investigated in this study perform similarly, with only small differences in some measures of visual performance and symptoms. In general, HOMCL seems to perform slightly better for near vision and ADT1 slightly better for distance vision. However, there are many exceptions to this general trend and there were a number of wearers with strong preferences for each type. The most successful clinicians will be those who are prepared to try more than one brand to determine the optimally performing MFCL for each individual patient.

## CRediT authorship contribution statement

**Bruce JW. Evans:** Writing – review & editing, Writing – original draft, Visualization, Validation, Supervision, Software, Project administration, Methodology, Investigation, Funding acquisition, Formal analysis, Data curation, Conceptualization. **Robert Yammouni:** Writing – review & editing, Writing – original draft, Visualization, Validation, Supervision, Software, Project administration, Methodology, Investigation, Formal analysis, Data curation. **Fabrizio Zeri:** Writing – review & editing, Writing – original draft, Visualization, Validation, Supervision, Software, Project administration, Methodology, Investigation, Funding acquisition, Data curation, Conceptualization. **Silvia Tavazzi:** Writing – review & editing, Validation, Investigation. **Giulia Carlotta Rizzo:** Writing – review & editing, Validation, Investigation, Data curation. **Bo Lauenborg:** Writing – review & editing, Validation, Supervision, Software, Project administration, Methodology, Investigation, Funding acquisition, Data curation, Conceptualization. **Rasmus Hagenau:** Writing – review & editing, Validation, Investigation. **Amanda Wittendorf:** Writing – review & editing, Validation, Investigation. **Manuela Eckert Andersen:** Writing – review & editing, Validation, Investigation. **Dimple Shah:** Writing – review & editing, Supervision, Software, Resources, Project administration, Methodology, Conceptualization. **Natalia Vlasak:** Writing – review & editing, Supervision, Software, Resources, Project administration, Methodology, Conceptualization.

## Data availability

The de-identified dataset are available at the Mendeley data repository:

https://data.mendeley.com/datasets/4472mb9wnn/1

## Funding sources

This study was funded by HOYA Vision Care.

## Declaration of competing interest

The study was funded by HOYA Vision Care. Dimple Shah and Natalia Vlasak are employees of HOYA Vision Care.
