# Supplementary material for: Randomised controlled trial of HOYA one-day multifocal contact lenses: The HOMCL trial
Source: Heliyon. 2024 Nov 8;10(22):e40137. doi: 10.1016/j.heliyon.2024.e40137 (PMC11615493; doi:10.1016/j.heliyon.2024.e40137)
Supplement: Multimedia component 1 [file mmc1.pdf]

2 simple steps to fitting HOYA one day multifocal contact lenses

Step 1. Using binocularly balanced, vertex corrected best sphere apply trial HOYA one day multifocal lenses. Select the add power at least 0.50D less than the spectacle ADD.

**Age range**	**Recommended ADD**
40–50	+1.00
50–60	+1.50
60–70+	+2.00

Allow to settle for at least 15 mins.

2 simple steps to fitting HOYA one day multifocal contact lenses

Step 2. To evaluate distance and near vision:

- Assess binocular vision using real-world examples such as a mobile phone, PC screen and looking out of the window.

Distance vision enhancement:

1. Try a lower ADD power for both eyes
2. If the patient is trialling +1.00 ADD power, then using a trial frame, binocularly over-refract using -0.25DS step trial lenses until the distance and near vision is optimised.

Near vision enhancement:

1. Using a trial frame, binocularly over refract using +0.25DS steps until the distance and near vision is optimised.
2. If the distance vision is impacted, then try a higher ADD power

HOYA one day contact lenses

MULTIFOCAL

HOYA 1 DAY silicone hydrogel				MULTIFOCAL
**Base Curve**	**Diameter**	**Power Ranges**	**ADDs**	**Colour**
8.60	14.20mm	+5.00D to -6.00D<br>(in 0.25D steps)<br><br>-6.50D to -10.00D<br>(in 0.50D steps)	+1.00<br>+1.50<br>+2.00	Light HOYA Blue<br>handling tint

Material: sorafilcon A **Water Content:** 48% **DK/t:** 140@ -3.00D **Centre thickness:** 0.08mm @ -3.00D (variable with power)

Wearing schedule: Daily disposable

Recommended lens care: The lenses should be discarded after a single use. Not intended to be cleaned.
