# Supplementary figures and images for: Randomised controlled trial of HOYA one-day multifocal contact lenses: The HOMCL trial
Source: Heliyon. 2024 Nov 8;10(22):e40137. doi: 10.1016/j.heliyon.2024.e40137 (PMC11615493; doi:10.1016/j.heliyon.2024.e40137)

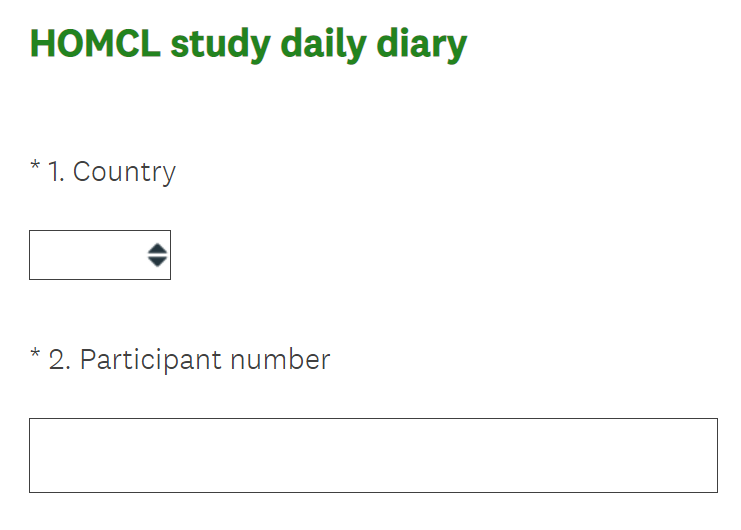


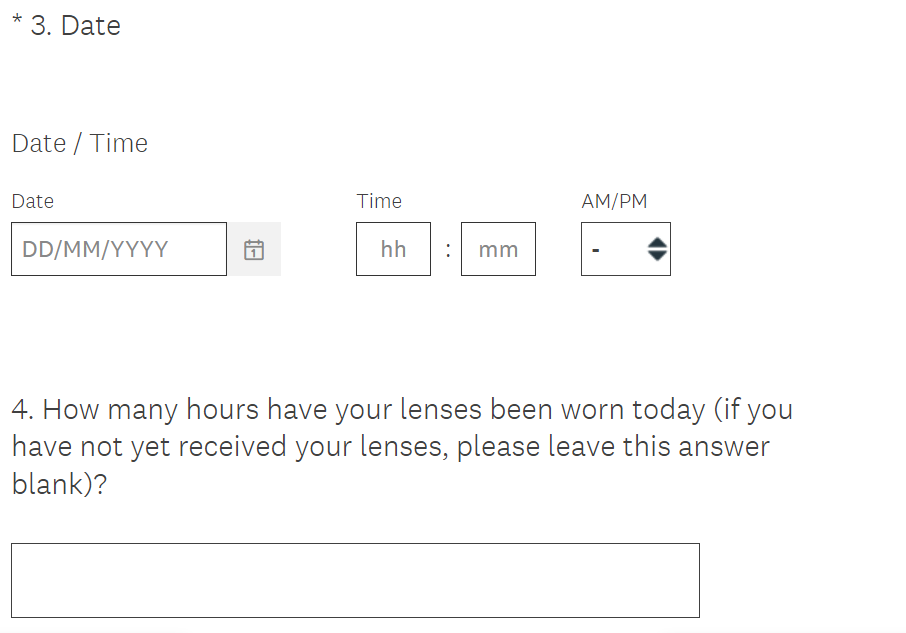


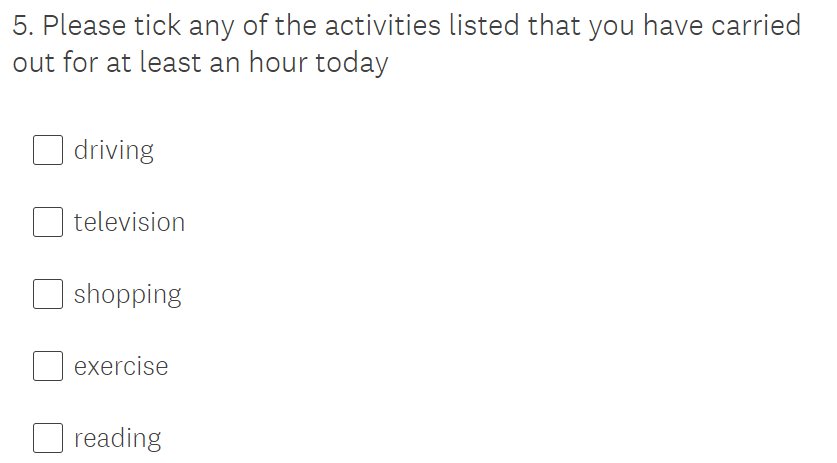


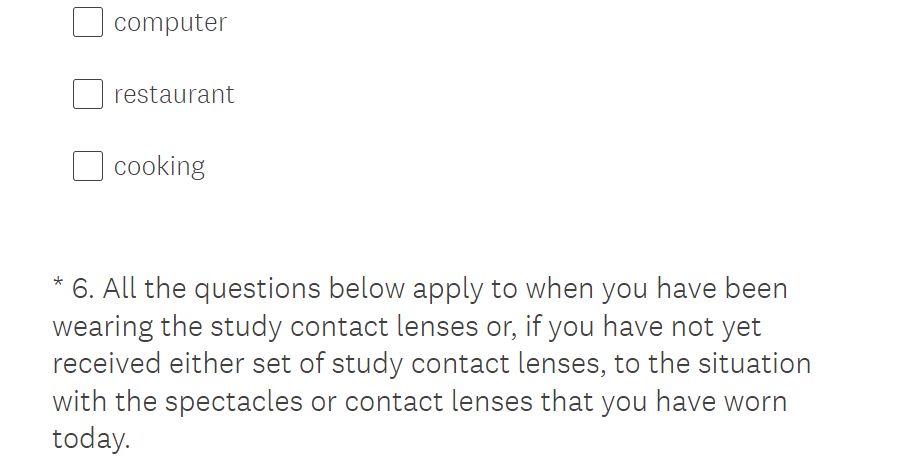


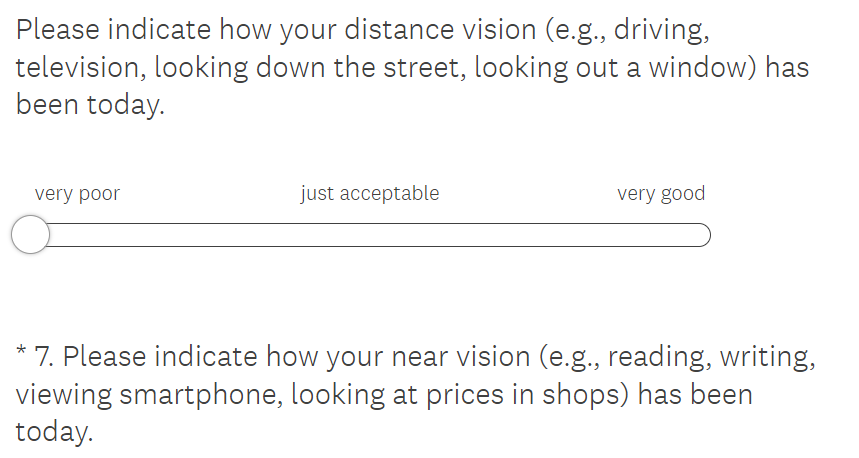


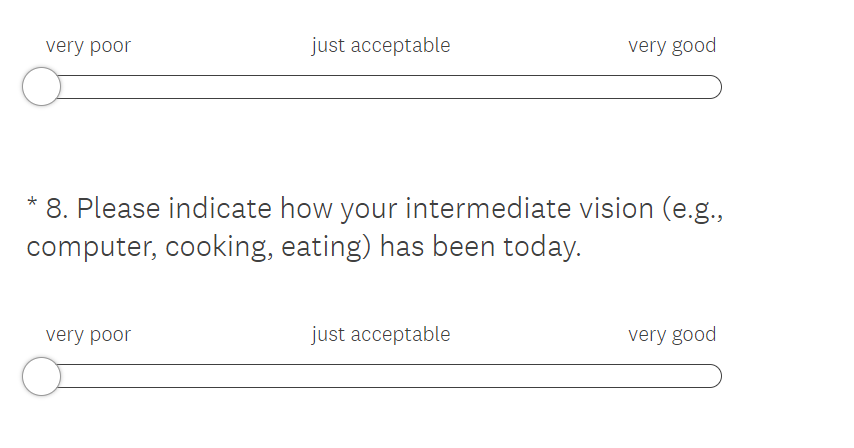


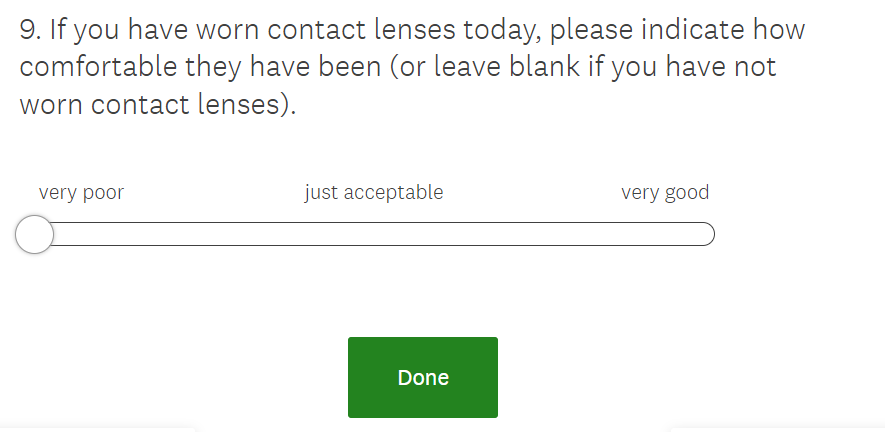

Supplement: Multimedia component 2 [file mmc2.docx]
